# Excitatory neuronal PLCG2 correlates with tau pathology in Alzheimer's disease via the alteration of the autophagy‐lysosomal pathway

**DOI:** 10.1002/alz70855_104168

**Published:** 2025-12-26

**Authors:** Tae Yeon Kim, Diana M Acosta, Nicholas Sweeney, Marie‐Amandine Bonte, Qi Guo, Ethan Rose, Jean Paul Chadarevian, Qin Ma, Hayk Davtyan, Mathew Blurton‐Jones, Hongjun Fu

**Affiliations:** ^1^ Ohio State University, Columbus, OH, USA; ^2^ The Ohio State University, Columbus, OH, USA; ^3^ University of California Irvine, Irvine, CA, USA; ^4^ Institute for Memory Impairments and Neurological Disorders, University of California Irvine, Irvine, CA, USA

## Abstract

**Background:**

Recent Genome‐Wide Association Studies (GWAS) have identified novel rare coding variants (P522R, M28L variants) of the enzyme phospholipase‐C‐γ2 (PLCG2) in late‐onset Alzheimer's Disease (LOAD). PLCG2 is a well‐known transmembrane‐associated enzyme, functioning downstream of immune receptors such as TREM2 in LOAD. It is reported that the PLCG2 P522R variant plays a protective role in AD and longevity by enhancing Aβ clearance and modulating microglial function. The role of PLCG2 and its variants in AD has been largely investigated in microglia and responses to Aβ pathology; however, little is known about the role of PLCG2 and its AD variants in other cell types or its role in tau pathology. PLCG2 is also a well‐known second messenger that activates the phosphorylation of GSK3β, which may lead to decreased tau hyperphosphorylation and promotion of the autophagy‐lysosomal pathway (ALP). ***Thus, we hypothesize that deficiency of excitatory neuronal PLCG2 enhances tau aggregation and propagation in AD by inhibiting the ALP***.

**Method:**

To evaluate the immunoreactivity of PLCG2 and its relationship with tau pathology, we performed western blot and immunofluorescence staining on human post‐mortem brains and PS19 tau mice. By stereotaxic injection of AAV8‐CaMKIIa‐cre and AD TBS‐soluble tau seeds in PLCG2‐floxed mice, we quantified tau spreading induced by PLCG2 knockdown. To further identify the role of the PLCG2 P522R variant (gain‐of‐function) in a complex human brain‐like environment, we generated human neural organoids derived from human iPSCs of a healthy donor and a CRISPR/Cas9‐edited PLCG2 P522R knock‐in (KI) cell line. To investigate ALP dynamics induced by PLCG2, we used FUW‐mCherry‐GFP‐LC3 reporter assay in P522R KI organoids.

**Result:**

We found higher global protein levels of PLCG2 in human AD cases and PS19 mice, but pathological tau‐positive cells had a lower level of PLCG2 than neighboring pathological tau‐negative cells. Knockdown of PLCG2 in excitatory neurons increased tau spreading in PLCG2‐floxed mice. Autophagy flux increased in P522R KI organoids, even in the presence of Bafilomyacin‐A1 (autophagy inhibitor), compared to wild‐type organoids.

**Conclusion:**

These results suggest that repression or dysfunction of PLCG2 may contribute to tau pathology in excitatory neurons of AD, probably via the regulation of the ALP.

